# Tin Oxide (SnO_2_) Nanoparticles: Facile Fabrication, Characterization, and Application in UV Photodetectors

**DOI:** 10.3390/nano12040632

**Published:** 2022-02-14

**Authors:** Zhenping Huang, Jun Zhu, Yi Hu, Yueping Zhu, Guanghua Zhu, Lanping Hu, You Zi, Weichun Huang

**Affiliations:** 1 Nantong Normal College, Nantong 226010, China; rghzp@163.com (Z.H.); 2015001@ntnc.edu.cn (Y.Z.); 2 School of Chemistry and Chemical Engineering, Nantong University, Nantong 226019, China; 2007320020@stmail.ntu.edu.cn (J.Z.); 2107320023@stmail.ntu.edu.cn (Y.H.); zgh@ntu.edu.cn (G.Z.); hlp@ntu.edu.cn (L.H.)

**Keywords:** tin oxide, metal oxide, nanomaterials, calcination, photodetection

## Abstract

Tin oxide (SnO_2_) nanomaterials are of great interest in many fields such as catalytic, electrochemical, and biomedical applications, due to their low cost, suitable stability characteristics, high photosensitivity, etc. In this contribution, SnO_2_ NPs were facilely fabricated by calcination of tin (II) oxalate in air, followed by a liquid-phase exfoliation (LPE) method. Size-selected SnO_2_ NPs were easily obtained using a liquid cascade centrifugation (LCC) technique. The as-obtained SnO_2_ NPs displayed strong absorption in the UV region (~300 nm) and exhibited narrower absorption characteristics with a decrease in NP size. The as-fabricated SnO_2_ NPs were, for the first time, directly deposited onto a poly(ethylene terephthalate) (PET) film with a regular Ag lattice to fabricate a flexible working electrode for a photoelectrochemical (PEC)-type photodetector. The results demonstrated that the SnO_2_-NP-based electrode showed the strongest photoresponse signal in an alkaline electrolyte compared with those in neutral and acidic electrolytes. The maximum photocurrent density reached 14.0 μA cm^−2^, significantly outperforming black phosphorus nanosheets and black phosphorus analogue nanomaterials such as tin (II) sulfide nanosheets and tellurene. The as-fabricated SnO_2_ NPs with relatively larger size had better self-powered photoresponse performance. In addition, the as-fabricated SnO_2_-NP-based PEC photodetector exhibited strong cycling stability for on/off switching behavior under ambient conditions. It is anticipated that SnO_2_ nanostructures, as building blocks, can offer diverse availabilities for high-performance self-powered optoelectronic devices to realize a carbon-neutral or carbon-free environment.

## 1. Introduction

A variety of strategies have been proposed over the last three decades to address critical global issues such as energy shortages and the impacts of a carbon-free environment [1,2,3,4,5]. Optoelectronic devices based on semiconducting materials have shown promising potential for utilizing abundant solar energy [6,7,8,9]. To date, a great deal of research has focused on the rational fabrication of high-performance semiconducting nanomaterials such as two-dimensional (2D) black phosphorus (BP) and its analogues [10,11], 2D tellurene [12], 0D or 2D selenium (Se) nanomaterials [13,14], etc., for their excellent optoelectronic device performances. For example, in 2017, Zhang et al. [11] successfully fabricated large-sized BP nanosheets (NSs) using a facile liquid-phase exfoliation (LPE) method, and these were employed as electrode materials to fabricate self-powered photodetectors displaying comparable photoresponse activity and environmental robustness under light illumination. In 2018, Ye et al. [15] successfully synthesized large-area, high-quality 2D tellurene NSs, with tunable thickness from a monolayer to tens of nanometers, and with lateral sizes of up to 100 µm, by a hydrothermal method. These exhibited high on/off ratios (10^6^), environmentally excellent stability, and carrier mobilities of about 700 cm^2^ V^−1^ s^−1^. Furthermore, in 2019, our group [6] rationally synthesized telluride–selenium (Te–Se) roll-to-roll nanotubes with different Se contents by epitaxial growth of Se on precursor Te nanotubes. These not only demonstrated a significantly improved capacity for self-powered photodetection but also remarkably enhanced the photocurrent density and stability in various aqueous electrolytes such as HCl, NaCl, and KOH solutions. However, the environmental instability of nanostructures (e.g., BP NSs and Se NSs) upon exposure to air, and the complex, time-consuming, and high-cost synthetic process make it difficult for them to satisfy the growing requirements of modern devices. Therefore, the exploitation of high-quality, cost-effective, and stable self-powered nanostructures is of great value for the performance optimization of optoelectronic devices.

It is well known that a standard photodetector should meet the “5S” requirement: high sensitivity, high signal-to-noise ratio, high spectral selectivity, high speed, and high stability [16,17]. Self-powered UV photodetectors play a vital role in many commercial and scientific applications including flame sensing, optical communication, satellite launching, biological and chemical analysis, and astronomical studies [18,19,20]. SnO_2_, a IV–VI semiconductor, is used as a UV detection material due to a variety of merits such as its suitable bandgap (3.6–4.0 eV) [7,21], high chemical stability, high electron mobility [22], etc. These characteristics make it suitable for many intriguing applications such as in batteries [23,24], sensors [25,26], catalysis [27,28], solar cells [22,29], water purification [7,30,31], and biomedical applications [32,33]. Moreover, the bandgap energy of SnO_2_ has great potential for bridging the bandgap space between BP (0.3–2.0 eV) [1,11], and hexagonal boron nitride (5.0–6.0 eV) [34]. These features of SnO_2_ make it a promising semiconducting material for practical application in self-powered UV photodetectors under ambient conditions, and it holds great promise in the field of renewable energy and renewable energy consumption for the achievement of carbon neutrality targets.

In this contribution, SnO_2_ nanostructures were facilely synthesized by calcination of tin (II) oxalate at 700 °C in air, and a combination of liquid-phase exfoliation (LPE) and liquid cascade centrifugation (LCC) was carried out to prepare size-selected SnO_2_ NPs. The as-prepared SnO_2_ NPs had an average diameter of 92 nm, 78 nm, and 56 nm as the centrifugation speed in the LCC process was increased. All the SnO_2_ NPs displayed strong absorptionn in the UV region (~300 nm) with relatively narrower absorption characteristics with a decrease in NP size. The as-fabricated SnO_2_ NPs were, for the first time, directly deposited onto a poly(ethylene terephthalate) (PET) film with a regular Ag lattice, to fabricate a flexible electrode for a photoelectrochemical (PEC)-type photodetector. The photoresponse results for this PEC-type photodetector demonstrated that it not only showed the strongest photoresponse signal in an alkaline electrolyte compared with those in neutral and acidic electrolytes, with maximum photocurrent density of 14.0 μA cm^−2^, but also displayed excellent self-powered photoresponse performance, as well as high stability in an alkaline electrolyte. Due to the facile fabrication, tunable UV absorption, excellent self-powered response and high environmental stability, it is expected that the present work can provide fundamental guidance on self-powered PEC photodetectors based on SnO_2_ nanostructures and inspire more research interest in next-generation devices, to achieve carbon neutrality targets.

## 2. Experimental Section

### 2.1. Materials

Tin (II) oxalate powder (99.9%), isopropyl alcohol (IPA, 99.9%), dimethyl formamide (DMF, 99.9%), poly(vinylidene fluoride) (PVDF, *M*_n_ = 71,000 g mol^−1^), and dimethyl formamide (DMF, 99.9%)were purchased from Sigma-Aldrich, Shanghai, China and used as received. PET film with a regular Ag lattice was purchased from IVTech-Jiangsu Co., Ltd., Nantong, Jiangsu, China. Hydrochloric acid (HCl, 38%), potassium chloride (KCl, 99.9%), and potassium hydroxide (KOH, 99.9%) were purchased from Alfa Aesar, Haverhill, MA, USA and used as received. Double-distilled deionized water was used for the preparation of the aqueous electrolyte.

### 2.2. Fabrication of SnO_2_ NPs

SnO_2_ nanostructures were facilely synthesized by calcination of tin (II) oxalate powder in air at 600 °C and 700 °C for a predetermined time (4 h and 8 h). Then, the as-obtained SnO_2_ nanostructures were dispersed into IPA solvent with a concentration of 12 mg mL^−1^ for sonication overnight at room temperature. An LCC technique was employed to collect different sizes of SnO_2_ NPs. For convenience, the SnO_2_ NPs collected at the centrifugation speed ranges of 2000–3000 rps, 5000–6000 rps, and 9000–10,000 rps were abbreviated as SnO_2_ NPs-1, SnO_2_ NPs-2, and SnO_2_ NPs-3. If not specified, SnO_2_ NPs denotes that they were obtained at a centrifugation speed range of 2–10 k.

### 2.3. Characterization

X-ray diffraction (XRD) patterns were obtained using a D8 Discover 25 X-ray diffractometer (Bruker) with a Cu K (k = 1.54056 Å) radiation source at room temperature collected from 5° to 85°. The morphology and dimension of the SnO_2_ nanostructures were determined by scanning electron microscopy (SEM, JSM-6701F, JEOL) and transmission electron microscopy (TEM, FEI F30, 300 kV) at an acceleration voltage of 5.0 kV with sample sputtering applied before analysis. The atomic arrangement of the as-fabricated SnO_2_ NPs was determined by high-resolution TEM (HRTEM). The SnO_2_ NPs sample was loaded onto ultrathin carbon-coated holey copper support films with 300-mesh copper grids for TEM measurements. UV–Vis absorbance spectrometry (Cary 60, Agilent, Santa Clara, CA, USA) with a spectral range of 200–1100 nm was performed to record UV–Vis–NIR absorption spectra of all the fabricated SnO_2_ NPs at room temperature. A typical photoresponse behavior was studied using a PEC measurement system [35,36]. The three-electrode system consisted of a working electrode (SnO_2_ NPs deposited on a clean PET film with a regular Ag lattice, photoanode), a counter electrode (platinum wire, photocathode), and a reference electrode (a saturated calomel electrode), together with aqueous electrolytes (0.05 M HCl, 0.05 M KCl, and 0.05 M, 0.10 M, and 0.50 M KOH). Amperometric current–time (*I*–*t*) curves were recorded at bias voltages of −0.4 V, −0.2 V, and 0 V under laser irradiation with different power densities (Table S1). Electrochemical impedance spectra (EIS) were collected at an amplitude of 0.005 V in the frequency range from 1 to 10^5^ Hz.

## 3. Results and Discussion

**Figure 1:**
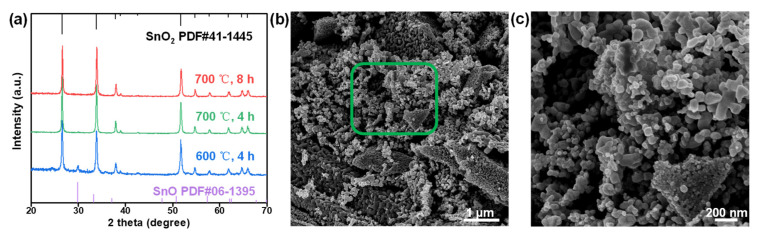
Structural characterization of the SnO_2_ nanostructures. (**a**) XRD patterns of the sample prepared by calcination of SnC_2_O_4_ at different temperatures for a predetermined time in air. (**b**) SEM image of SnO_2_ nanostructures obtained by calcination of SnC_2_O_4_ at 700 °C for 8 h in air and (**c**) the enlarged area corresponding to the region surrounded by a green box in (**b**).

In this work, SnO_2_ nanostructures were fabricated by facile calcination on a large scale at relatively high temperature. The XRD results in Figure 1a show that only a high calcination temperature and a long calcination time, e.g., 700 °C for 8 h, could efficiently facilitate the formation of pure SnO_2_ structures. However, low calcination temperature or a short calcination time, e.g., calcination at 700 °C for 4 h or 600 °C for 8 h could lead to the formation of SnO structures to some extent, as shown by the appearance of the peak at ~30° caused by the insufficient decomposition of tin (II) oxalate powder, which can be indexed to the characteristic peak of SnO (Figure 1a) [37,38]. It can be observed from the SEM image that relatively uniform SnO_2_ nanostructures were obtained (Figure 1b), with diameters ranging from ~20 nm to ~150 nm (Figure 1c).

**Figure 2:**
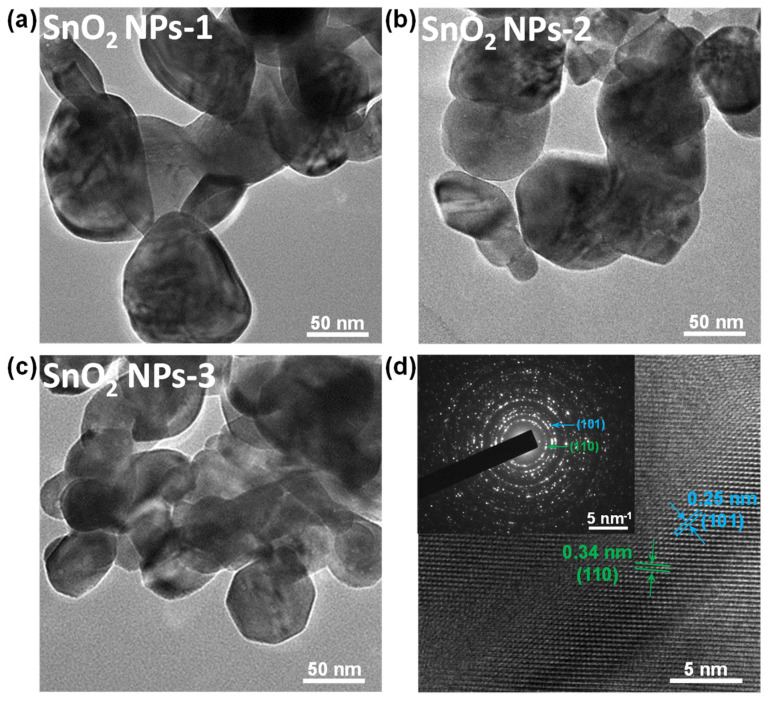
TEM characterization of the SnO_2_ NPs with different sizes: (**a**) SnO_2_ NPs-1, (**b**) SnO_2_ NPs-2, (**c**) SnO_2_ NPs-3. (**d**) HRTEM image; inset shows its SAED pattern.

In order to obtain size-selected SnO_2_ NPs, a combination of LPE and LCC techniques was used. The LPE technique was used to exfoliate the SnO_2_ nanostructures calcined at 700 °C for 8 h, and the LCC technique was used to collect the as-exfoliated SnO_2_ NPs with a more uniform size. As shown in Figure 2, the TEM image shows that the SnO_2_ NPs obtained by a combination of LPE and LCC techniques displayed clear size selection, i.e., SnO_2_ NPs-1 collected at a centrifugation speed range from 2000 rps to 3000 rps had an average diameter of ~92 nm (Figure 2a); SnO_2_ NPs-2 collected at a centrifugation speed range from 5000 rps to 6000 rps had an average diameter of ~78 nm (Figure 2b); and SnO_2_ NPs-3 collected at a centrifugation speed range from 9000 rps to 10,000 rps had an average diameter of ~56 nm (Figure 2c). The HRTEM image shows clear lattice fringes of 0.25 nm and 0.34 nm (Figure 2d), which can be assigned to the (101) and (110) planes of the SnO_2_ crystals, respectively [29,39]. The selected-area electron diffraction (SAED) pattern (Figure 2d, inset) further confirms the formation of the SnO_2_ nanostructures.

**Figure 3:**
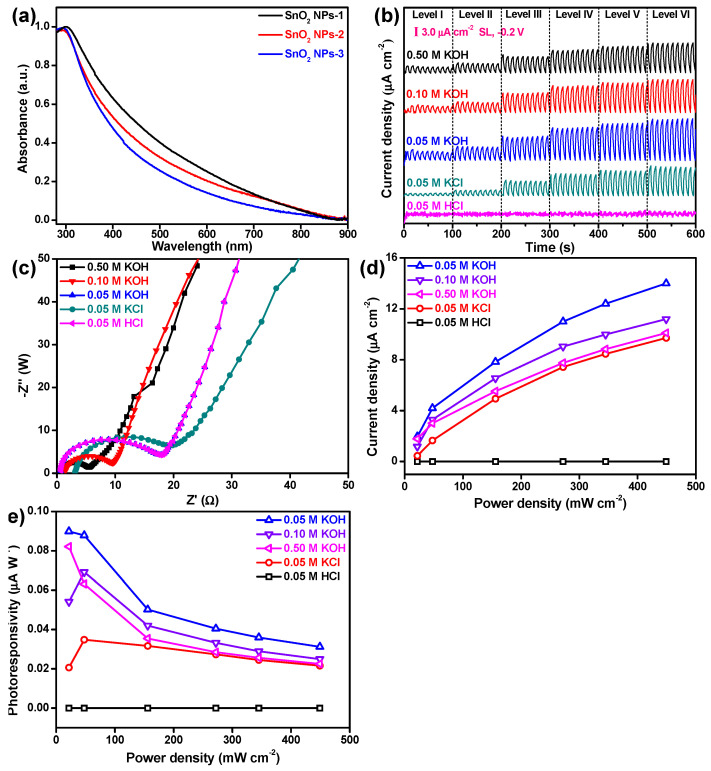
Photoresponse behavior. (**a**) UV–Vis–NIR spectra of the size-selected SnO_2_ NPs; (**b**) on/off switching behavior of the SnO_2_-NPs-1-based electrode in different electrolytes under SL at −0.2 V; (**c**) current density of the SnO_2_-NPs-1-based electrode as a function of power density; (**d**) interface resistances between the SnO_2_-NPs-1-based electrode and different electrolytes; (**e**) photoresponsivity of the SnO_2_-NPs-1-based electrode as a function of power density.

UV–Vis–NIR absorption spectroscopy was employed to characterize the optical response of the size-selected SnO_2_ NPs, as shown in Figure 3a. It can be seen that all the three samples displayed a relatively narrow optical absorption, but with an increase in size of the SnO_2_ NPs the absorption became wider and stronger, in good agreement with the results reported for tin (II) sulfide (SnS) nanosheets [40] and tungsten disulfide (WS_2_) nanosheets [41]. Moreover, simulated light (SL, 300–2000 nm, Table S1) was employed as incident light to illuminate the SnO_2_-NP-based electrode for electricity detection. As shown in Figure 3b,c, except for the acidic electrolyte condition, the electrode always exhibited a gradually increasing photoresponse signal with an increase in the power density (*P_λ_*) of SL in alkaline or neutral electrolyte, indicating that the performance optimization of this SnO_2_-NP-based photodetector can be easily realized. The influence of the electrolyte on the photoresponse behavior was also studied, as shown in Figure 3b,c. It can be observed that the alkaline electrolyte was the most competitive electrolyte in this SnO_2_-NP-based photodetector, because the highest photocurrent density (*I* = (*I*_on_ − *I*_off_)/*S*) was delivered in 0.05 M KOH compared with those delivered in 0.05 M KCl and 0.05 M KCl. Here, *I*_on_ and *I*_off_ denote the photocurrent with and without irradiation, respectively, and *S* represents the effective area of the SnO_2_ NPs under irradiation. This could be mainly attributable to the different functionalities of the SnO_2_ NPs on the surface and the electrolytes, due to the similar resistance interface (*R*) between the SnO_2_ NPs and the electrolytes (*R*_0.05M KOH_ = 17.6 Ω, *R*_0.05M KCl_ = 18.3 Ω, and *R*_0.05M HCl_ = 17.6 Ω), as seen in Figure 3d. It is noted that the highest *I* in this work reached 14.0 μA cm^−2^, significantly outperforming black phosphorus nanosheets (0.382 μA cm^−2^) [11] and black phosphorus analogue nanomaterials such as tin (II) sulfide nanosheets (1.59 μA cm^−2^) [40] and tellurene (0.365 μA cm^−2^) [12] (Table S2). Furthermore, the electrolyte concentration also played an important role in the photoresponse behavior. It can be observed from Figure 3b,c that the lower the KOH concentration, the higher the value of *I*, which could be mainly ascribed to the *R* (*R*_0.50M KOH_ = 5.5 Ω, *R*_0.10M KOH_ = 8.7 Ω, and *R*_0.05M KOH_ = 17.6 Ω), as seen in Figure 3d, indicating that at the studied electrolyte concentrations, high electrolyte concentration severely suppressed the efficient separation of hole (*h*^+^)–electron (*e*^−^) pairs. In addition, unlike for *I*, larger photoresponsivity (*R* = *I*/*P_λ_*) was usually obtained under relatively low *P_λ_*, especially in KOH electrolytes (0.05 M, 0.10 M, and 0.50 M), as seen in Figure 3e. The highest *R* value reached 0.10 μA W^−1^, which is comparable to those of black phosphorus nanosheets (2.2 μA W^−1^) [11] and tellurene (3.0 μA W^−1^) [12]. It should be noted that the much larger *P_λ_* of SL employed in this work leads to low values of *R* compared to those reported in other studies [6,42].

**Figure 4:**
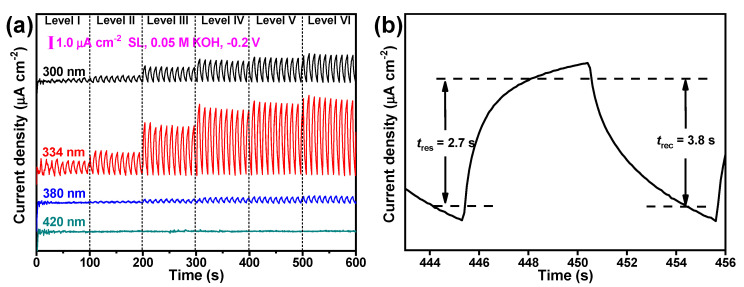
(**a**) The influence of laser wavelength on the on/off switching behavior of the as-fabricated SnO_2_ NPs at −0.2 V and (**b**) response time and decay time.

In order to emphasize the specific response behavior in the UV region, incident lasers with different wavelengths (300, 334, 380, and 420 nm) were used to irradiate the SnO_2_-NP-based electrodes. As shown in Figure 4a, the as-prepared SnO_2_-NP-based electrode displayed significantly the largest *I* under the 334 nm laser, compared with the values under 300, 380, or 420 nm lasers under the same conditions, verifying that this SnO_2_-NP-based photodetector has a specific UV photodetection performance. The largest *I* and *R* values, observed under the 334 nm laser at an external voltage of −0.2 V, were 5.35 μA cm^−2^ and 4.61 μA W^−1^, respectively. Note that this specific UV photodetection performance under a 300 nm or 334 nm laser does not dramatically deteriorate when the external voltage changes remarkably (see Figure S1). The response time (*t*_res_) and recovery time (*t*_rec_) are usually assigned to the time interval for the rise and decay from 10% to 90% and from 90% to 10% of the peak value, respectively [40]. It can be seen in Figure 4b and Figure S2 that the *t*_res_/*t*_rec_ of the as-fabricated SnO_2_-NP-1-based electrode was 2.7 s/3.8 s, close to those of the SnO_2_-NP-2-based electrode (3.6 s/3.9 s) and the SnO_2_-NP-3-based electrode (3.5 s/3.8 s), all of which are comparable to those of SnSe nanosheets (1.2 s/2.2 s) [43] and bismuth selenide nanosheets (5.3 s/9.5 s) [44].

**Figure 5:**
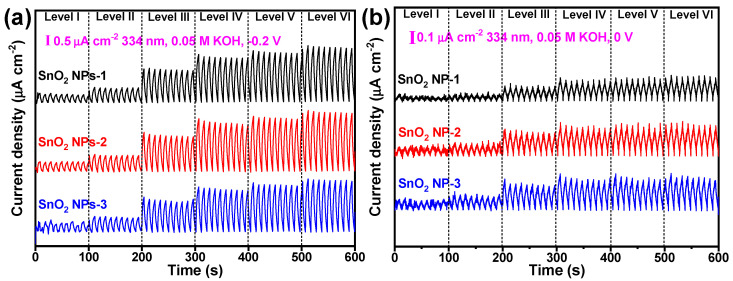
Effect of SnO_2_ NPs with different sizes on the photoresponse behavior at external voltages of (**a**) −0.2 V and (**b**) 0 V, under SL.

Due to the size-dependent absorption of the SnO_2_ NPs as mentioned above and seen in Figure 2a, the photoresponse behavior and self-powered photoresponse performance (zero external voltage) were also studied, as shown in Figure 5. It can be seen from Figure 5a and Figure S3 that the photocurrent densities of SnO_2_ NPs-2 and SnO_2_ NPs-3 irradiated by SL (Figure 5a) and the 334 nm laser (Figure S3) at an applied exteral voltage were almost the same under the same conditions, while they were evidently superior to that of SnO_2_ NPs-1, indicating that relatively smaller SnO_2_ NPs were more beneficial for strengthening the PEC signal. Similarly, at zero external voltage, the self-powered photoresponse signal had the same trend as at −0.2 V (Figure 5b), and the largest self-powered *I* was 2.25 μA cm^−2^, which is also better than that of black phosphorus nanosheets (0.265 μA cm^−2^) [11] and selenium quantum dots (0.0385 μA cm^−2^) [14]. In addition, the reduction in *I* after one month was calculated to be only 27.6% (Table S2 and Figure S4), and the observed on/off switching behavior was unchanged from the beginning. This, combined with almost the same color before and after measurement (Figure S5), confirmed that the as-prepared SnO_2_-NP-based electrode shows excellent photoresponse stability and holds great potential for practical applications in optoelectronic devices.

## 4. Conclusions

In this contribution, SnO_2_ NPs were facilely fabricated by calcination of tin (II) oxalate in air, followed by a combination of LPE and LCC techniques. The XRD patterns showed that the SnO_2_ nanostructures could be successfully obtained after sufficient calcination for 8 h at 700 °C. The SEM image showed that the as-fabricated SnO_2_ NPs had a particle size ranging from ~20 nm to ~150 nm. The PEC result demonstrated that the SnO_2_-NP-based electrode not only had a strong electrolyte-dependent PEC performance (i.e., the best performance in a low-concentration alkaline electrolyte), but also exhibited size-modulated photoresponse behavior, i.e., the smaller the size, the better the PEC performance. Moreover, the SnO_2_-NP-based photodetector showed a much stronger and more stable PEC signal in the UV region (300 nm and 334 nm), compared to that in the visible region (420 nm), indicating that it has great potential as a building block in UV photodetectors. In addition, the excellent PEC stability even for a one-month PEC measurement makes the SnO_2_-NP-based electrode a promising candidate for practical applications. It is anticipated that the present work can provide fundamental guidance on the performance of a PEC-type SnO_2_-NP-based photodetector and offer extensive availabilities for high-performance SnO_2_-based heterostructures for constructing next-generation optoelectronic devices.

## Data Availability

The data presented in this study are available on request from the corresponding author.

## Supplementary Materials

The following supporting information can be downloaded at: https://www.mdpi.com/article/10.3390/nano12040632/s1, Table S1: Laser power density (Pλ) of of incident light with various irradiation wavelengths; Table S2: The comparison of the SnO_2_ NP-based PEC electrodes and other published PEC electrodes; Figure S1: The influence of laser wavelength on the on/off switching behavior of the as-fabricated SnO_2_ NPs at −0.4 V; Figure S2: The response time and decay time of (a) the as-fabricated SnO_2_ NP-2 and (b) SnO_2_ NP-3-based electrodes irradiated by SL in 0.05 M KOH: Figure S3: Size effect of the SnO_2_ NPs with different sizes on the photoresponse behavior at external voltages of −0.4 V under 334 nm laser; Figure S4: (a) Photoresponse stability of the as-prepared SnO_2_ NPs-1, and (b) its enlarged area; Figure S5: The pictures of (a) the as-prepared SnO_2_ NP-1 powder and (b) SnO_2_ NP-1-based electrode.
