# Supplementary material for: Tin Oxide (SnO2) Nanoparticles: Facile Fabrication, Characterization, and Application in UV Photodetectors
Source: Nanomaterials (Basel). 2022 Feb 14;12(4):632. doi: 10.3390/nano12040632 (PMC8876611; doi:10.3390/nano12040632)
Supplement: Supplementary file 1 [file nanomaterials-12-00632-s001.zip › nanomaterials-1533210-supplementary.pdf]

Tin Oxide Nanoparticles: Facile Fabrication, Characterization and Application in UV Photodetectors

Zhenping Huang [1], Jun Zhu [2], Yi Hu [2], Yueping Zhu [1], Guanghua Zhu [2], Lanping Hu [2], You Zi [2,*] and Weichun Huang [2,*]

[1] Nantong Normal College, Nantong 226010, China; rghzp@163.com (Z.H.); 2015001@ntnc.edu.cn (Y.Z.)
[2] School of Chemistry and Chemical Engineering, Nantong University, Nantong 226019, China; 2007320020@stmail.ntu.edu.cn (J.Z.); 2107320023@stmail.ntu.edu.cn (Y.H.); zgh@ntu.edu.cn (G.Z.); hlp@ntu.edu.cn (L.H.)
*Correspondence: ziyou@ntu.edu.cn (Y.Z.); huangweichun@ntu.edu.cn (W.H.)

Table S1 Laser power density (P_λ) of of incident light with various irradiation wavelengths.

Laser	P_λ (mW cm^{-2})					
	Level I	Level II	Level III	Level IV	Level V	Level VI
Simulated light (SL)	21.8	47.7	156	272	345	449
300 nm	0.15	0.20	0.30	0.55	0.75	0.84
334 nm	0.15	0.30	0.85	1.50	1.95	2.30
380 nm	0.35	0.55	2.10	3.60	4.74	5.95
420 nm	4.25	9.85	32.7	56.6	71.9	94.7

Table S2 The comparison of the SnO_2 NP-based PEC electrodes and other published PEC electrodes.

Materials	Experimental conditions	Photocurrent density (I)/ μA cm^{-2}	Response time (t_{res})/ Recovery time (t_{rec})	Reduction of I after one month	Ref.
SnO_2 NPs	0.05-0.50 M KOH	14.0	2.7 s/3.8 s	27.6%	This work
Black phosphorus Nanosheets (NSs)	0.05-1.0 M KOH and 0.5 M Na_2SO_4	0.382	0.5 s/1.1 s	~82%	1
Te NSs	0.1-1.0 M KOH	0.365	-	~24%	2
Se QDs	0.5 M KOH	1.80	0.02 s/0.62 s	~48%	3
SnS NSs	0.1-1.0 M Na_2SO_4, 0.1-0.5 M H_2SO_4, and 0.1-0.5 M KOH	1.59	0.1-0.3 s/0.3-0.5 s	45.3%-62.7%	4
Bi quantum dots	0.1-1.0 M KOH and 0.1-0.5 M Na_2SO_4	1.02	0.1 s/0.2 s	56.7%	5

[1] Ren, X.; Li, Z.; Huang, Z.; Sang, D.; Qiao, H.; Qi, X.; Li, J.; Zhong, J.; Zhang, H. Environmentally Robust Black Phosphorus Nanosheets in Solution: Application for Self-Powered Photodetector. *Adv. Funct. Mater.* **2017**, *27*, 1606834.

[2] Xie, Z.; Xing, C.; Huang, W.; Fan, T.; Li, Z.; Zhao, J.; Xiang, Y.; Guo, Z.; Li, J.; Yang, Z.; Dong, B.; Qu, J.; Fan, D.; Zhang, H. Ultrathin 2D Nonlayered Tellurium Nanosheets: Facile Liquid-Phase Exfoliation, Characterization, and Photoresponse with High Performance and Enhanced Stability. *Adv. Funct. Mater.* **2018**, *28*, 1705833.

[3] Jiang, X.; Huang, W.; Wang, R.; Li, H.; Xia, X.; Zhao, X.; Hu, L.; Chen, T.; Tang, Y.; Zhang, H. Photocarrier Relaxation Pathways in Selenium Quantum Dots and Their Application in UV-Vis Photodetection. *Nanoscale* **2020**, *12*, 11232–11241.

[4] Huang, W.; Xie, Z.; Fan, T.; Li, J.; Wang, Y.; Wu, L.; Ma, D.; Li, Z.; Ge, Y.; Huang, Z.N.; Dai, X.; Xiang, Y.; Li, J.; Zhu, X.; Zhang, H. Black-Phosphorus-Analogue Tin Monosulfide: An Emerging Optoelectronic Two-Dimensional Material for High-Performance Photodetection with

Improved Stability under Ambient/Harsh Conditions. *J. Mater. Chem. C* **2018,** *6*, 9582–9593.
[5] Xing, C.; Chen, X.; Huang, W.; Song, Y.; Li, J.; Chen, S.; Zhou, Y.; Dong, B.; Fan, D.; Zhu, X.; Zhang, H. Two-Dimensional Lead Monoxide: Facile Liquid Phase Exfoliation, Excellent Photoresponse Performance, and Theoretical Investigation. *ACS Photonics* **2018**, *5*, 5055–5067.

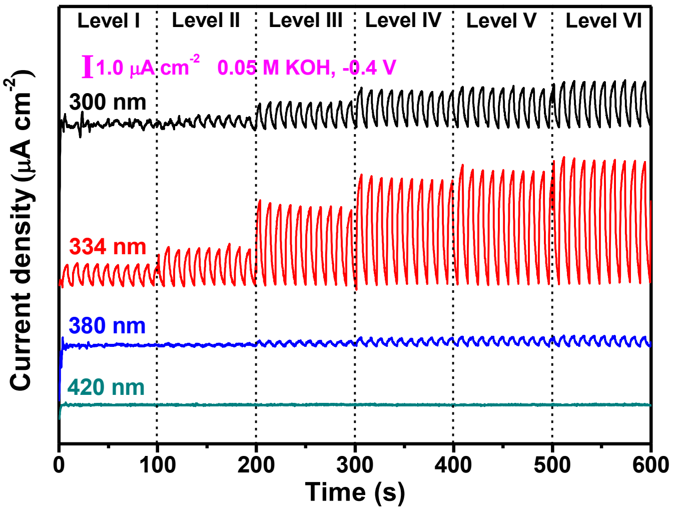

Figure S1. The influence of laser wavelength on the on/off switching behavior of the as-fabricated SnO_2 NPs at -0.4V.

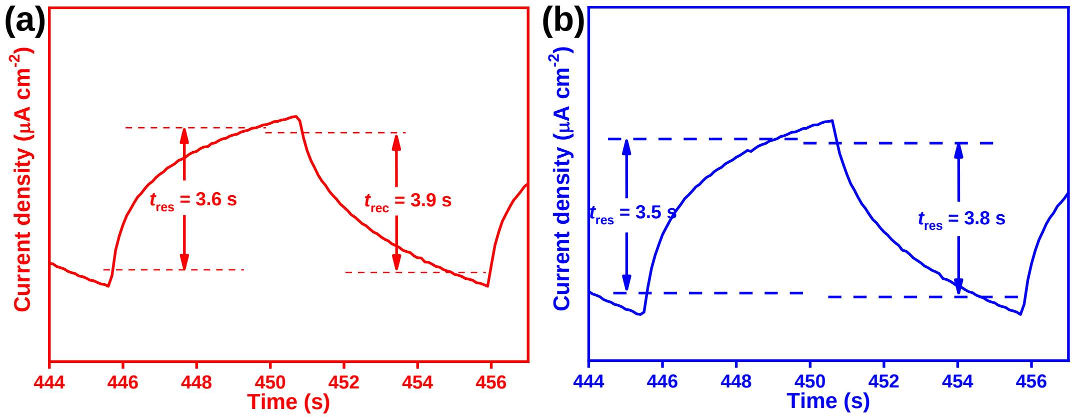

Figure S2. The response time and decay time of (a) the as-fabricated SnO_2 NP-2 and (b) SnO_2 NP-3-based electrodes irradiated by SL in 0.05 M KOH.

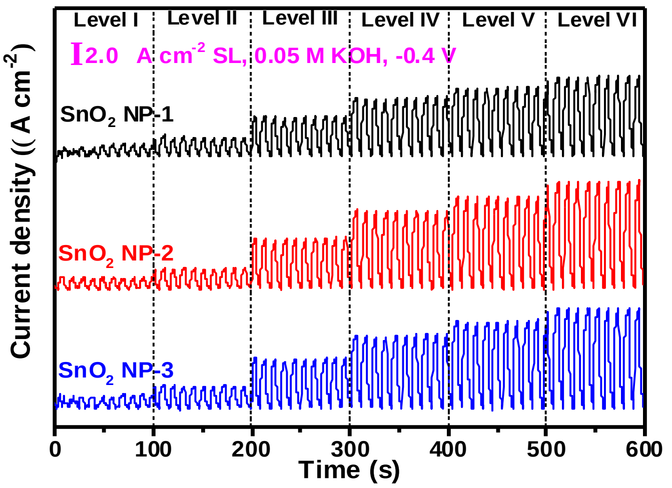

Figure S3. Size effect of the SnO_2 NPs with different sizes on the photoresponse behavior at external voltages of -0.4 V under 334 nm laser.

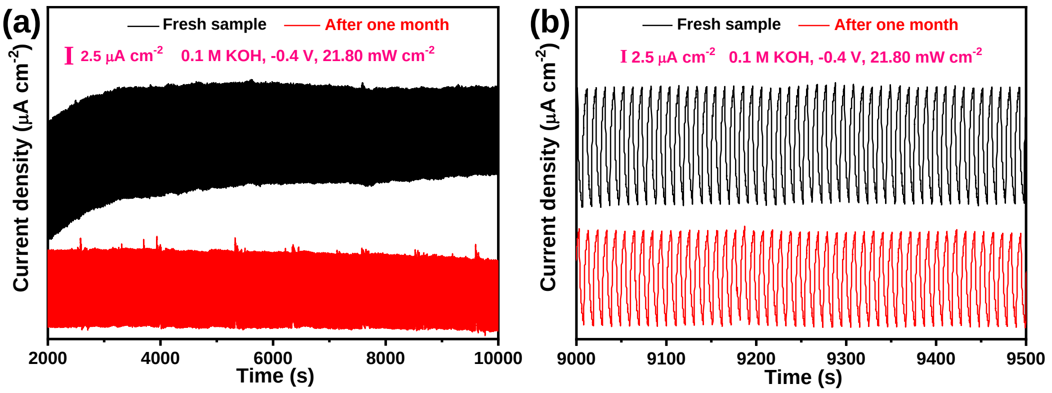

Figure S4. (a) Photoresponse stability of the as-prepared SnO_2 NPs-1, and (b) its enlarged area.

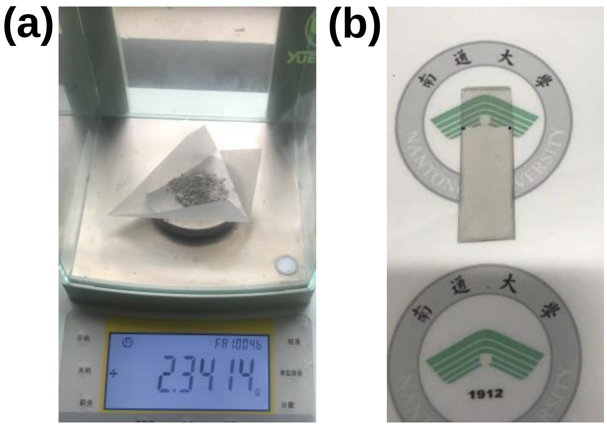

Figure S5. The pictures of (a) the as-prepared SnO_2 NP-1 powder and (b) SnO_2 NP-1-based electrode.
